# Dysregulation of MicroRNA-152-3p is Associated with the Pathogenesis of Pulpitis by Modulating SMAD5

**DOI:** 10.3290/j.ohpd.b4132867

**Published:** 2023-06-05

**Authors:** Fengyang Yu, Pengyue Wang, Guoliang Gong

**Affiliations:** a Attending Physician, Department of Orthodontics, Perfect Dental Care, Hangzhou, China. Study design, conducted the experiment and analysed the data, wrote the manuscript, reviewed and approved the final manuscript.; b Attending Physician, Department of Orthodontics, Renxin Dental, Ningbo, China. Study design, conducted the experiment and analysed the data, reviewed and approved the final manuscript.; c Professor, Department of Orthodontics, Dr. Art & Smile Dental Care, Hangzhou, China. Study design, conducted the experiment and analysed the data, revised the manuscript, reviewed and approved the final manuscript.

**Keywords:** diagnostic value, human dental pulp cells, miR-152, pulpitis, SMAD

## Abstract

**Purpose:**

To research the role of microRNA (miR)-152 in the pathogenesis of pulpitis using a cell model based on human dental pulp cells (HDPCs) treated with lipopolysaccharides (LPS).

**Materials and Methods:**

The biological activity of HDPCs infected by LPS was measured using a cell counting kit (CCK-8), Transwell test, flow cytometry, and fluorescent quantitative PCR. The concentration of superoxide dismutase (SOD) and malondialdehyde (MDA) was evaluated using an assay kit, the levels of interleukin (IL)-1β and IL-6 were measured by enzyme-linked immunosorbent assay (ELISA), and the targeting relationship between SMAD5 and miR-152 was measured by the double-luciferase report test. The expression of cell cycle-related CyclinD1 and BAX was assessed by PCR. By plotting a receiver operating characteristic (ROC) curve, the diagnostic value of miR-152 was shown.

**Results:**

The level of miR-152 in HDPCs induced by LPS decreased, while the level of SMAD5 increased. After overexpressing miR-152 in LPS-induced HDPCs, the viability was elevated, the apoptosis rate decreased, CyclinD1 was elevated, BAX diminished, the inflammatory cytokines (IL-6 and IL-1β) were inhibited, the activity of SOD increased, and the MDA content decreased. miR-152 targeted regulation of SMAD5, and SMAD5 modulated the effects of miR-152 on cell viability, apoptosis, inflammation, and the oxidative response of HDPCs. Reduced miR-152 expression was verified in patients with pulpitis, which could be a biomarker for pulpitis.

**Conclusion:**

miR-152 was found to be a biomarker correlated with the pathogenesis of pulpitis and the biological behaviour of HDPCs.

Pulpitis is considered as a complcation of dental caries,^21^ with a complicated pathogenesis and development. It is a non-specific inflammatory response and a specific immune response caused by bacteria and their metabolites infecting dental pulp tissue.^33^ Pulpitis refers to inflammatory lesions occurring in the pulp tissue of the teeth and is mainly caused by dental infection.^14^ The main clinical manifestations include severe pain, which often makes eating and sleeping difficult; it seriously affects work and social interactions as well as emotion status, thus endangering physical and mental health and quality of life.^12^ Pulpitis is the most common pathological disorder affecting the dental pulp tissue, and can lead to pulp necrosis and loss of vitality of the tooth, brittle dentin, increased propensity to fracture, tooth loss, and loss of the immune defense response of the pulp.^4^ Root canal therapy is the most common treatment.^31^ Endodontically treated teeth are compromised by sensory loss, increased fragility, a tendency to split, and tooth discolouration.^1^ Therefore, it is necessary to explore the aetiology and pathogenesis of pulpitis, find therapeutic targets, achieve early inflammation control, block the progression of inflammation, promote pulp regeneration, and restore pulp vitality.

miRNA is involved in changing the expression genes involved in pulpitis.^15,26^ According to one study, let-7c-5p, miR-410, and miR-146a are related to oral diseases, such as dental pulp inflammation and pulpitis.^22^ miR-18b-5p has an interactive relationship with lncRNA DUXAP8 in pulpitis, which induces apoptosis, inflammation, and oxidation in human dental pulp cells (HDPCs).^5^ miR-27a-3p is highly expressed in HDPCs that contain lipopolysaccharides (LPS) and may promote inflammation.^25^ miR-152 is important for normal cell function, and participates in the occurrence, development, outcome, and other pathological processes of diseases. In inflamed pulps, the relative quantity of miR-152 decreased, indicating that this change may promote inflammatory lesions of dental pulp tissues.^36^ Additionally, miR-152 regulates cell cycle progression and resists cellular senescence of H_2_O_2_-damaged human dental pulp stem cells.^6^ Thus, we hypothesise that miR-152 may play a regulatory role in the pathogenesis of pulpitis.

Thus, the purpose of this study was to elucidate the role of microRNA (miR)-152 in the pathogenesis of pulpitis using a cell model based on human dental pulp cells (HDPCs) infected with lipopolysaccharides (LPS). Additionally, we collected pulp tissues from teeth with pulpitis, detected the expression of miR-152, and investigated the effect of miR-152 in the clinic.

## Materials and Methods

### Patients and Collection of Clinical Samples

This study was approved by the Medical Ethics Committee of Dr. Art & Smile Dental Care. All patients signed informed consent before sample collection. Seventy-seven patients with a diagnosis of pulpitis and who had undergone tooth extraction at Dr. Art & Smile Dental Care participated. Eighty pulp tissues were extracted from eighty persons without pulpitis and served as the control group. All control teeth were healthy and free of caries. Pulpitis-tissue inclusion criteria were: donors aged between 18 and 60 years old; teeth were diagnosed with acute or chronic pulpitis by endodontists according to clinical examination and imaging examination, and the pulp could be removed in its entirety. The patients had not taken any medications for 3 months prior to their participation in the study. Pulp tissue samples from patients with serious diseases of the heart, brain, liver, kidney, and endocrine system were excluded. Forty-one (41) males and 40 females comprised the control group. The average age was 37.6 ± 12.0 years in controls and 38.3 ± 13.0 years in the pulpitis group. The BMI was 22.7 ± 1.4 kg/m^2^ in the control group and 22.7 ± 1.4 in pulpitis patients. The pulpitis group included 36 males and 40 females. No significant differences were observed in age, BMI, or sex (p > 0.05, Table 1). Seventy-two patients also exhibited caries and 10 periodontitis, but no individual was found to have both caries and periodontitis (p < 0.01, Table 1).

**Table 1:** Basic clinical data of the subjects

Items	Control group (n = 77)	Pulpitis group (n = 80)	Significance (p)
Age (years)	37.62±11.94	38.30±13.02	0.74
BMI (kg/m^2^)	22.66±1.35	22.68±1.40	0.92
**Sex**			0.68
Male	41	36	
Female	40	40	
**Caries**			
Yes	0	72	<0.01
No	77	8	
**Periodontitis**			
Yes	0	10	<0.01
No	77	70	

All data are presented as mean ± standard deviation or n.

Teeth were immersed in normal saline for 30 min. After cleaning the root surface of any remaining periodontal tissue, the root tips were cut off, and the pulp was removed and stored in liquid nitrogen for 6 months.

### Establishment of Cell Models and Transfection

HDPCs from ATCC (Manassas, VA, USA) were exposed to the bacterial endotoxin LPS (derived from *E. coli* 0111:B4, Sigma-Aldrich; St Louis, MO, USA) at a concentration of 1 µg/ml for 24 h. After that, the cells were extracted and prepared for subsequent experiments.

HDPCs that had grown to 80% confluency were rinsed with PBS (Sigma-Aldrich), digested with trypsin (Sigma-Aldrich), and resuspended. The cells were counted using a cell counter and seeded into six-well plates, and the cell density was adjusted to 8 x 10^4^/ml. A solution was prepared of synthesised miRNA powder (Sangon; Shanghai, China) with enzyme-free water at a concentration of 20 nmol/l in an ultra-clean bench, then mixed and stored in a refrigerator at -20°C for 24 h. Lipofectamine 3000 reagent (Thermo Fisher Scientific; Waltham, MA, USA) was obtained for the cell transfection protocol, and the following experiments were carried out after 48 h of transfection.

The expression of miR-152 and SMAD5 mRNA was detected by the real-time fluorescence quantitative PCR method.

Total RNA was extracted using an RNA fast tissue/cell kit (TIANGEN; Beijing, China) on a clean table as per kit instructions. For miRNA, synthesised cDNA was obtained using a miRNA 1st-strand cDNA-synthesis kit (Vazyme; Nanjing, China). For other genes, cDNA was synthesised by transcribing RNA according to the reverse transcription kit instructions (TaKaRa; Kusatsou, Japan). The StepOnePlus real-time PCR system (Applied Biosystems; Foster City, CA, USA) was employed using a SYBR Green qPCR kit (biosharp; Hefei, China). U6 and GAPDH were quantified as internal references. The relative expression was analysed using the 2-∆∆CT formula.

### Cell Viability Detected by CCK-8

HDPCs were inoculated into 96-well plates. On the 1st, 2nd and 3rd days, the original culture medium was siphoned off, and 10 µl CCK-8 reagent (Beyotime; Shanghai, China) was supplemented and incubated for 4 h. The absorbance value was obtained with an enzyme label (450 nm) from BIO-RAD (Hercules, CA, USA).

### Apoptosis Phenomenon Detected by Flow Cytometry

The cells were collected, annexin V-fluorescein isothiocyanate (FITC)/propidium iodide (PI) was added and left to react for 15 min. A LSRFortessa flow cytometer (BD Biosciences Pharmigen; San Diego, CA, USA) was used to detect the cells. The percentage of annexin V-positive cells was calculated as the percentage of apoptosis. All the reagents were obtained from BD Biosciences Pharmigen. Flow Jo software (Tree Star; Ashland, OR, USA) was employed to analyse the data collected.

### Enzyme-linked Immunosorbent Assay (ELISA)

The HDPCs in a good growth state were cultured on 6-well plates at a density of 1x10^5^ cells per well. The original culture medium was discarded when the cells adhered to the wall, and the cell supernatant was collected. The levels of TNF-α, IL-6, and IL-8 were detected by ELISA, and the kit instructions (Sangon; Shanghai, China) were strictly followed. 100 μl of standard working solution and the HDCP sample were added to each reaction well and incubated at 37°C after sealing the plate. 100 μl of biotin-labeled antibody was added to each reaction well and placed in an incubator at 37°C after sealing the plate. HRP-labeled streptavidin was also added to this mixture and incubated at 37°C after sealing the plate. A chromogenic agent was added to each reaction well, and the plate was sealed at about 37°C to prevent exposure to light. Terminating solution was added to each reaction well, and the OD value was measured with an enzyme-labeling instrument (Varioskan LUX, Thermo Fisher Scientific; Waltham, MA, USA) at 450 nm wavelength.

### Detection of Superoxide Dismutase (SOD) and Malondialdehyde (MDA) Levels

The cell supernatant was collected. The detection of SOD and MDA was performed strictly following the instructions of the total SOD assay kit with WST-8 and MDA assay kit (Beyotime). For detection of SOD, the samples were mixed with 160 µl of WST-8/enzyme working reagent and 20 µl of working solution, and were then incubated for 30 min. The absorbance data at 450 nm were recorded. The MDA-detection working fluid was added to samples for MDA measurement, and the mixture was heated in boiling water for 15 min. After a water bath, the plates were cooled to room temperature and centrifuged at 1000 g for 10 min. Supernatant was gathered and absorbance was subsequently measured at 532 nm using a Sunrise microplate reader (Tecan Austria; Grodig, Austria).

### Reporter Gene Assay

An online database for prediction of miRDB targets predicted a binding region between miR-152 and SMAD5. Recombinant vector plasmids of wild type (WT)-SMAD5 and mutant (MUT)-SMAD5, miR-152 (negative control) NC, miR-152 mimics, and miR-152 inhibitors were all aquired from GenePharma (Shanghai, China). The carriers were co-transfected with synthetic nucleotide sequences of miR-152 into 293T cells. The ratio of fluorescence value of firefly luciferase to that of renilla luciferase in cells was measured by double-luciferase activity detection reagent (Yeason; Shanghai, China).

### Statistical Analysis

Graphpad 6.0 (Graphpad Software; San Diego, CA, USA) was used to conduct one-way and two-way ANOVAs. In the cell experiments, each group of cells was set up with 5 multiple wells. SPSS 20.0 software package (IBM; Armonk, NY, USA) was used to perform the receiver operating characteristic (ROC) analysis. Statistical significance was set at p < 0.05.

## Results

### miR-152 Reversed Viability and Apoptosis Induced by LPS

**Fig 1:**
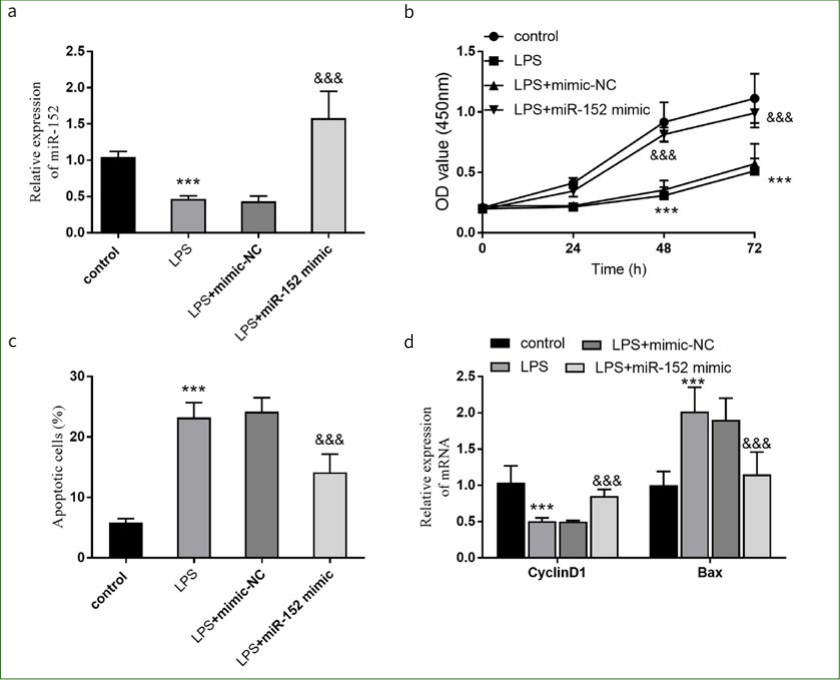
Impacts of miR-152 on viability and apoptosis. a. Transfection of mimic reversed the decrease of miR-152 expression. b. LPS damaged the cell viability and miR-152 ameliorated the impacts of LPS. c. Effect of miR-152 on apoptosis of HDPCs induced by LPS. d. Levels of CyclinD1 and Bax were improved by miR-152. ***p < 0.001, relative to control group; &&&p < 0.001, relative to LPS group. NC: negative control.

As shown by PCR, the expression of miR-152 in the cells treated with LPS was lower than that in control cells (p < 0.001, Fig 1a). This decreased expression changed after transfection with miR-152 mimic (p < 0.001, Fig 1a). The cell viability at 48 h and 72 h was reduced in the LPS group, while the apoptosis rate was increased (all p < 0.001, Figs 1b and 1c). Higher miR-152 expression increased cell viability and limited the apoptosis of HDPCs (all p < 0.001, Figs 1b and 1c). The pro-apoptotic BAX and cycle-associated CyclinD1 were estimated to reflect cell activity and apoptosis. CyclinD1 was suppressed and BAX was induced by the LPS treatment, but miR-152 reversed the partial impacts of LPS on HDPCs (p < 0.001, Fig 1d).

### Effect of miR-152 on LPS-induced Oxidative Stress and Inflammation

**Fig 2:**
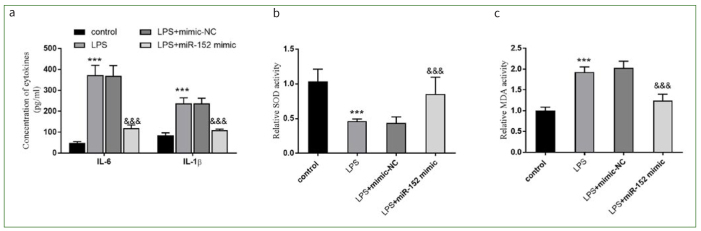
Effects of miR-152 on inflammation and oxidation. a. IL-6 and IL-1β were inhibitory by the miR-152 overexpression. b. and c. The oxidative status was reflected by SOD and MDA activity. ***p < 0.001, relative to control group; &&&p < 0.001, relative to LPS group. NC: negative control.

The impacts of miR-152 on HDPCs were further confirmed by the inflammation and oxidative response data. The levels of IL-6 and IL-1β in the LPS group were increased, while the release of these cytokines was inhibited in the LPS + miR-152 mimic group (p < 0.001, Fig 2a).

The activity of SOD in HDPCs was reduced and the amount of MDA was augmented (all p < 0.001, Figs 2b and 2c). SOD activity in the LPS + miR-152 mimic group was partially increased, while the MDA level had dropped (all p < 0.001, Figs 2b and 2c).

### miR-152 Targets SMAD5 Gene Expression

**Fig 3:**
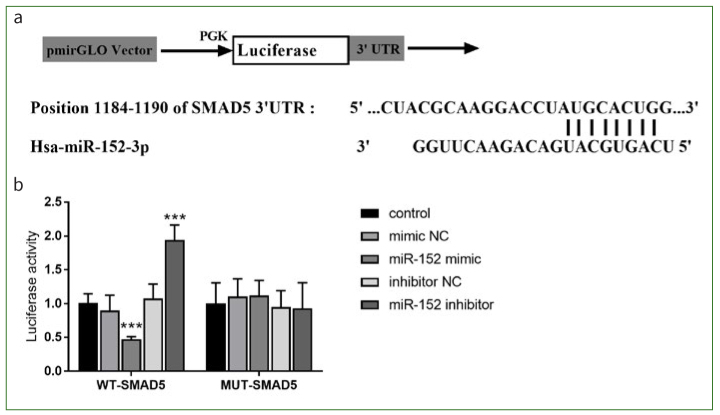
SMAD5 targets miR-152. a. Complementary nucleotide sequences of miR-152 and SMAD5. b. Decrease of luciferase activity after co-transfection of SMAD5 and miR-152. ***p < 0.001, relative to control group. NC: negative control.

As depicted in Fig 3a, miR-152 and SMAD5 had complementary nucleotide sequences. In the cells transfected with WT-SMAD5, the luciferase activity of the miR-152 mimic group was reduced and of miR-152 inhibitor group was enhanced (p < 0.001, Fig 3b). In cells transfected with MUT-SMAD5, all subgroups showed no statistically significant difference in luciferase activity (p > 0.05, Fig 3b).

### Effects of Overexpression of SMAD on Viability, Apoptosis, Inflammatory Cytokine Secretion, and Oxidation of HDPCs Stimulated by LPS

**Fig 4:**
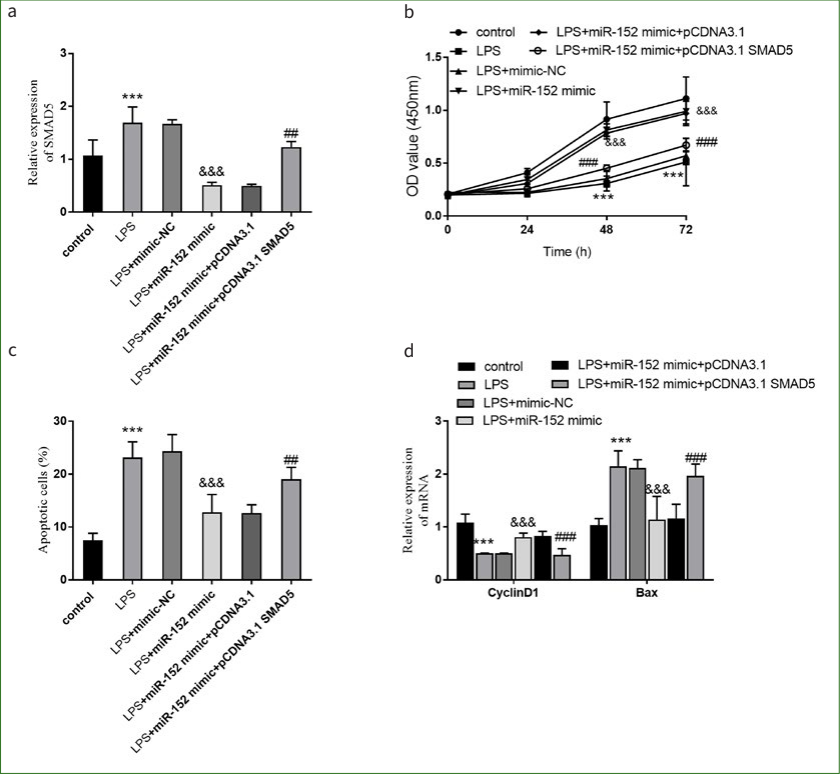
SMAD5 reversed the effects of miR-152 on viability and apoptosis of HDPCs damage induced by LPS. a. The success of transfecting pCDNA3.1 SMAD5. b. OD values at 450 nm. c. Apoptotic percentage of HDPCs. d. Change of CyclinD1 and Bax expression. ***p < 0.001, relative to control group; &&&p < 0.001, relative to LPS group; ##p < 0.01, ###p < 0.001, relative to LPS group. NC: negative control.

To further explore how miR-152 is involved in the regulation of trophoblast function and the related mechanism, we set up a group transfected with pCDNA3.1 SMAD5 and miR-152 mimics. The expression of SMAD5 was increased by LPS, while its expression was reduced by miR-152 overexpression (p < 0.001, Fig 4a). The treatment of pCDNA3.1 SMAD5 reversed the diminished expression of SMAD5 in the LPS + miR-152 group (p < 0.01, Fig 4a).

**Fig 5:**
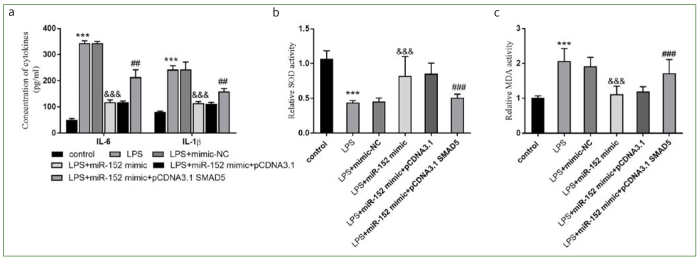
SMAD5’s effect on inflammation and oxidative stress. a. Inflammatory activators were improved by SMAD5. b. and c. SMAD5 modulated the oxidative stress. ***p < 0.001, relative to control group; &&&p < 0.001, relative to the LPS group; ##p < 0.01, ###p < 0.001, relative to the LPS group. NC: negative control.

The viability was statistically significantly increased in the LPS + miR-152 group, but decreased by the overexpression of SMAD5 (p < 0.001, Fig 4b). The overexpression of miR-152 limited the proportion of apoptotic cells, while the overexpression of SMAD5 increased it (p < 0.01, Fig 4c). Overexpression of miR-152 increased the amount of CyclinD1 and down-regulated the level of BAX, while the abundance of SMAD5 supressed the effects of miR-152 on HDPCs (p < 0.001, Fig 4d). Overexpression of miR-152 could attenuate the secretion of IL-6 and IL-1β, a tendency which was reversed by SMAD5 (p < 0.01, Fig 5A). While miR-152 inhibited oxidative stress, SMAD5 reversed the inhibitory effect of miR-152 (all p < 0.001, Figs 5b and 5c). Collectively, miR-152 could participate in the regulation of the activities of HDPCs by regulating the expression of SMAD5.

### RT-PCR Validation Analysis of miR-152 in Human Pulpitis

**Fig 6:**
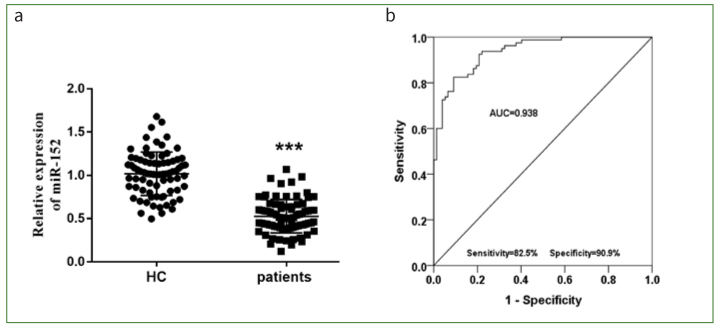
a. Alterations in quantity of miR-152 in patients. b. ROC curve was plotted to demonstrate the diagnostic value of miR-152.

Based on the abnormally expressed miR-152 found in-vitro, pulp tissue was taken from patients with pulpitis for miRNA detection. The expression of miR-152 in pulp tissues taken from these patients was diminished, indicating that pulpitis was associated with the decrease in miR-152 (p < 0.001, Fig 6a). The area under the curve (0.938) in Fig 6b demonstrates the efficacy of miR-152 in diagnosing pulpitis.

## Discussion

Pulp tissue is a loose connective tissue in human teeth,^2^ a highly differentiated mesenchymal tissue surrounded by hard dentin.^10^ As one of the most common oral maladies, pulpitis can be cause by plaque biofilms; it is also an opportunistic infection caused by oral microorganisms invading the dental pulp tissue.^13^ The pathogenesis of pulpitis involves the pathogenic stimulation of dental pulp by various physical and chemical factors, which leads to the destruction of dental pulp by bacteria.^18^ The main clinical symptoms are severe pain in teeth and gums. Without timely and effective intervention for patients with pulpal inflammation, the development of pulpitis may lead to the degeneration or necrosis of pulp tissue, cause periapical inflammation, alveolar bone defects, and other secondary diseases.^19^ Therefore, the study of the pathogenesis of pulpitis is necessary.

Dental pulp tissue produces defensive responses to eliminate infection.^27^ miRNAs are abnormally expressed in pulp tissues, and they participate in pulpitis by regulating related cell bio-function. miR-224-5p, miR-126, and miR-206 are associated with the pathogenesis of pulpitis by controlling the activities of dental pulp cells.^9,11,33^ miR-9 modulates the proliferation of HDPCs by targeting KLF5.^29^ miR-148a-3p modulates the viability and differentiation by the Wnt1/β-catenin axis.^16^ All these miRNAs are proven to be related to the development of pulpitis. miR-152 is a regulator in many disorders and is associated with the normal function of diverse types of cells. miR-152 can be involved in the development of thymic involution by suppressing the proliferative ability of thymic epithelial cells.^17^ miR-152 ameliorates the proliferation and CyclinD1 secretion of preadipocytes, thus participating in lipid accumulation.^7^ We used LPS to construct cell models, finding that miR-152 was upregulated in HDPCs treated by LPS. miR-152 reversed the adverse impacts of LPS on the viability and apoptotic percentage of HDPCs, implying that miR-152 was beneficial to the recovery of normal function of HDPCs. These impacts of miR-152 on cell viability were also reflected by the secretion of CyclinD1 and BAX. Inflammatory cytokines can regulate the inflammatory response of pulp cells at the molecular level, and participate throughout the progression of pulpitis.^30^ When pulpitis has systemic effects, the contents of IL-6, IL-1β, IL-10, and other cytokines in the body are significantly higher than those in the healthy pulp.^38^ Overexpression of miR-152 ameliorates the inflammatory factors; thus, it is associated with immunological regulation.^20^ The results of this study suggest that LPS could induce inflammation in HDPC, and upregulation of miR-152 expression could inhibit LPS-induced production of IL-6 and IL-1β in HDPC. In addition, miR-152 ameliorated the oxidative stress in HDPCs treated with LPS. It was concluded that miR-152 played an inhibitory role in inflammation and oxidation.

Further investigation by luciferase assay verified that miR-152 could negatively regulate the expression level of SMAD5. It is speculated that this targeting relationship may be related to the regulation of pulpitis by miR-152. SMAD5 regulates miR-135b in terms of odontoblast-like differentiation in HDPCs,^28^ indicating that SMAD5 may be correlated with pulpitis. Studies have shown that SMAD5 can activate the intracellular inflammatory response or related signaling pathways.^23^^,35^ Oxidative stress injury in macular degeneration is modulated by SMAD5, reflecting the association between SMAD5 and oxidation.^3^ The experimental results revealed that SMAD5 was elevated in the presence of LPS, and miR-152 inhibited SMAD5 expression in HDPCs. SMAD played a role in inhibiting viability and enhancing apoptosis, inflammation, and oxidation of HDPCs, which was closely related to the regulation of miR-152.

This investigation found reduced expression of miR-152 in the presence of pulpitis. Consistent with our findings, Zhong et al^36^ verified the downregulation of miR-152 in inflamed human pulps. The clinical significance of miR-152 has also been proven by previous studies. For instance, miR-152 is an abnormally expressed gene in prostate cancer. It may be used as a diagnostic tool in combination with miR-98-5p, miR-326, and miR-4289.^24^ Several miRNAs are found aberrantly expressed in pulpitis and may serve as biomarkers. miR-30b is overexpressed in tissue, plasma, and saliva samples of patients with pulpitis and correlates with inflammation.^34^ Zhou et al^37^ confirm that miR-27a-3p can serve to distinguish pulpitis from the pulp of healthy persons. As reflected in the ROC curve in this study, miR-152 might act as an indicator of patients with pulpitis, which can be used to target clinical treatment. As opposed to the study by Gu et al,^8^ the current study designed a model in which LPS treatment damaged the normal function of HDPCs, and focused on the function of miR-152 on cell viability, inflammation, apoptosis, and oxidative stress. Additionally, the diagnostic function of miR-152 was also evaluated in patients with pulpitis, which was not the case in the previous article.^8^

## Conclusion

Collectively, miR-152 could enhance viability and inhibit the inflammation, apoptosis, and oxidative stress of HDPCs induced by LPS. The mechanism of this effect was related to the fact that miR-152 targeted SMAD5. The expression of miR-152 was lower in patients with pulpitis and served as a biomarker in pulpitis. This investigation could provide a new basis for determining the aetiology and pathogenesis of pulpitis.

## References

[ref1] Batra R, Sisodiya M, Kumari P, Kaur S, Patil PB, Bhagat SK (2022). Fracture resistance to treated teeth using known endodontics techniques in Indian patients. Bioinformation.

[ref2] Chang HH, Chen IL, Wang YL, Chang MC, Tsai YL, Lan WC (2020). Regulation of the regenerative activity of dental pulp stem cells from exfoliated deciduous teeth (SHED) of children by TGF-β1 is associated with ALK5/SMAD2, TAK1, p38 and MEK/ERK signaling. Aging.

[ref3] Chen L, Ma B, Liu X, Hao Y, Yang X, Liu M (2020). H(2) O(2) induces oxidative stress damage through the BMP-6/SMAD/hepcidin axis. Dev Growth Differ.

[ref4] Clarkson JE, Ramsay CR, Mannocci F, Jarad F, Albadri S, Ricketts D (2022). Pulpotomy for the management of irreversible pulpitis in mature teeth (PIP): a feasibility study. Pilot Feas Stud.

[ref5] Dai Y, Xuan G, Yin M (2022). DUXAP8 promotes LPS-induced cell injury in pulpitis by regulating miR-18b-5p/HIF3A. Int Dent J.

[ref6] Dou W, Xie J, Chen J, Zhou J, Xu Z, Wang Z (2022). Overexpression of adrenomedullin (ADM) alleviates the senescence of human dental pulp stem cells by regulating the miR-152/CCNA2 pathway. Cell Cycle.

[ref7] Fan Y, Gan M, Tan Y, Chen L, Shen L, Niu L (2019). miR-152 regulates 3T3-L1 preadipocyte proliferation and differentiation. Molecules (Basel, Switzerland).

[ref8] Gu S, Ran S, Liu B, Liang J (2016). miR-152 induces human dental pulp stem cell senescence by inhibiting SIRT7 expression. FEBS Lett.

[ref9] Jiang L, Krongbaramee T, Lin X, Zhu M, Zhu Y, Hong L (2022). microRNA-126 inhibits vascular cell adhesion molecule-1 and interleukin-1beta in human dental pulp cells. J Clin Lab Anal.

[ref10] Kaviani N, Shahaboyi M, Khabazian A (2012). Determining the effect of implant surgery on blood oxygen saturation of the adjacent tooth. Dent Res J.

[ref11] Ke Z, Qiu Z, Xiao T, Zeng J, Zou L, Lin X (2019). Downregulation of miR-224-5p promotes migration and proliferation in human dental pulp stem cells. BioMed Res Int.

[ref12] Kearney M, Cooper PR, Smith AJ, Duncan HF (2018). Epigenetic approaches to the treatment of dental pulp inflammation and repair: opportunities and obstacles. Front Genet.

[ref13] Khorasani MMY, Hassanshahi G, Brodzikowska A, Khorramdelazad H (2020). Role(s) of cytokines in pulpitis: Latest evidence and therapeutic approaches. Cytokine.

[ref14] Kojima Y, Sendo R (2022). Maintaining tooth vitality with super minimally invasive pulp therapy. Cureus.

[ref15] Lei F, Zhang H, Xie X (2019). Comprehensive analysis of an lncRNA-miRNA-mRNA competing endogenous RNA network in pulpitis. PeerJ.

[ref16] Li Q, Huang L (2021). miR-148a-3p Regulates the invasion and odontoblastic differentiation of human dental pulp stem cells via the wnt1/β-catenin pathway. Int J Stem Cells.

[ref17] Li Y, Wang X, Wu Q, Liu F, Yang L, Gong B (2022). miR-152-3p Represses the proliferation of the thymic epithelial cells by targeting SMAD2. Genes.

[ref18] Liu B, Xiong M, Liu F, Chen W, Jiang S, Qu M (2022). Effect of enhanced recovery after surgery (ERAS) concept and cluster nursing on psychological state and pain of oral outpatients undergoing root canal therapy. Comput Math Meth Med.

[ref19] Liu M, Goldman G, MacDougall M, Chen S (2022). BMP Signaling pathway in dentin development and diseases. Cells.

[ref20] Liu X, Zhan Z, Xu L, Ma F, Li D, Guo Z (2010). MicroRNA-148/152 impair innate response and antigen presentation of TLR-triggered dendritic cells by targeting CaMKIIα. J Immunol (Baltimore, MD: 1950).

[ref21] López-Marcos JF (2004). Aetiology, classification and pathogenesis of pulp and periapical disease. Med Oral Patol Oral Cir Bucal.

[ref22] Maqbool M, Syed NH, Rossi-Fedele G, Shatriah I, Noorani TY (2022). MicroRNA and their implications in dental pulp inflammation: current trends and future perspectives. Odontol.

[ref23] Moonen JR, Chappell J, Shi M, Shinohara T, Li D, Mumbach MR (2022). KLF4 recruits SWI/SNF to increase chromatin accessibility and reprogram the endothelial enhancer landscape under laminar shear stress. Nature Communic.

[ref24] Moya L, Meijer J, Schubert S, Matin F, Batra J (2019). Assessment of miR-98-5p, miR-152-3p, miR-326 and miR-4289 Expression as biomarker for prostate cancer diagnosis. Int J Molec Sci.

[ref25] Nara K, Kawashima N, Noda S, Fujii M, Hashimoto K, Tazawa K (2019). Anti-inflammatory roles of microRNA 21 in lipopolysaccharide-stimulated human dental pulp cells. J Cell Physiol.

[ref26] Sehic A, Tulek A, Khuu C, Nirvani M, Sand LP, Utheim TP (2017). Regulatory roles of microRNAs in human dental tissues. Gene.

[ref27] Song H, Lei Y, Xing Z, Liu M (2022). Minocycline plus zinc oxide eugenol cement might be a promising alternative for acute pulpitis. Evid Based Complementary Altern Med. eCAM.

[ref28] Song Z, Chen LL, Wang RF, Qin W, Huang SH, Guo J (2017). MicroRNA-135b inhibits odontoblast-like differentiation of human dental pulp cells by regulating SMAD5 and SMAD4. Int Endod J.

[ref29] Wang JH, He DE (2021). Simvastatin treatment promotes proliferation of human dental pulp stem cells via modulating PI3K/AKT/miR-9/KLF5 signalling pathway. J Cell Molec Med.

[ref30] Wu Q, Li S, Li R, Chen X, Guo L, Zheng Y (2022). The detection of pro-inflammatory cytokines in exudates from dental pulp tissues. Cytokine.

[ref31] Xu LJ, Zhang JY, Huang ZH, Wang XZ (2022). Successful individualized endodontic treatment of severely curved root canals in a mandibular second molar: A case report. World J Clinical Cases.

[ref32] Zhang B, Huo S, Cen X, Pan X, Huang X, Zhao Z (2020). circAKT3 positively regulates osteogenic differentiation of human dental pulp stromal cells via miR-206/CX43 axis. Stem Cell Res Ther.

[ref33] Zhang L, Bai L, Ren Q, Sun G, Si Y (2018). Protective effects of SIRT6 against lipopolysaccharide (LPS) are mediated by deacetylation of Ku70. Molec Immunol.

[ref34] Zhang N, Zhang Q, Yang W, Miao L, Wang N, Wei S (2019). Decreased expression of microRNA-30b promotes the development of pulpitis by upregulating the expression of interleukin-6 receptor. Exp Ther Med.

[ref35] Zhang X, Ai F, Li X, She X, Li N, Tang A (2015). Inflammation-induced S100A8 activates Id3 and promotes colorectal tumorigenesis. Int J Cancer.

[ref36] Zhong S, Zhang S, Bair E, Nares S, Khan AA (2012). Differential expression of microRNAs in normal and inflamed human pulps. J Endod.

[ref37] Zhou M, Li C (2021). Clinical value and potential target of miR-27a-3p in pulpitis. Neuroimmunomodul.

[ref38] Zhu N, Wang D, Xie F, Qin M, Lin Z, Wang Y (2020). Fabrication and characterization of calcium-phosphate lipid system for potential dental application. Front Chem.

